# The Intracardiac Masses That Wouldn’t Melt: Wrong Diagnosis or Wrong Treatment — A Case Report

**DOI:** 10.1016/j.jscai.2026.104398

**Published:** 2026-03-25

**Authors:** Zaran A. Butt, Adam Freegrove, Laura Murphy, Niall Mahon, Ivan Casserly, Andrew S.P. Sharp, Ronan Margey

**Affiliations:** a Department of Cardiology, Mater Misericordiae University Hospital, Dublin, Ireland; b Department of Histopathology, Mater Misericordiae University Hospital, Dublin, Ireland; c University College Dublin School of Medicine, Health Sciences Centre, Dublin, Ireland

**Keywords:** case report, embolectomy, mechanical aspiration, right atrial mass, thrombectomy, thrombus

## Abstract

A 64-year-old woman with biventricular cardiomyopathy and a cardiac resynchronization device developed recurrent spontaneous sub-segmental pulmonary emboli. Large mobile intracardiac masses adherent to all three leads were detected and failed to regress with sequential trials of antithrombotic therapy. Surgical intervention was deemed high-risk. The masses were successfully aspirated percutaneously using the AlphaVac F1885 percutaneous mechanical aspiration system (AngioDynamics, Inc) under trans-esophageal and fluoroscopic guidance, which is the first reported use of this recently CE-marked device for extracting large right atrial masses in Europe. There was no evidence of recurrent masses at thirty-day follow-up. Histopathological analysis confirmed organized thrombi.

## Case presentation

**Figure 1:**
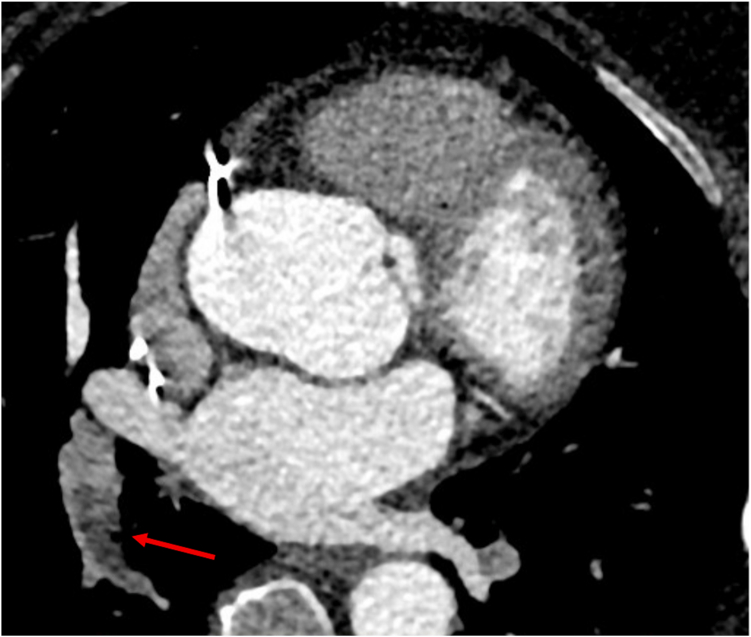
**Computed tomography pulmonary angiogram demonstrating a partially occlusive filling defect (red arrow) within the distal right intralobar artery, extending into a medial segmental branch of right lower lobe artery.**

A 64-year-old woman with bicuspid aortopathy, obesity (body mass index 36 kg/m^2^), and prior implantation of a cardiac resynchronization therapy device for nonischemic biventricular cardiomyopathy had an incidental segmental pulmonary embolus detected on routine surveillance aortic computed tomography (CT) performed 2 months after a mild COVID-19 illness (Figure 1). She received a 6-month course of direct oral anticoagulation therapy with complete resolution of emboli on the 6-month follow-up CT.

**Figure 2:**
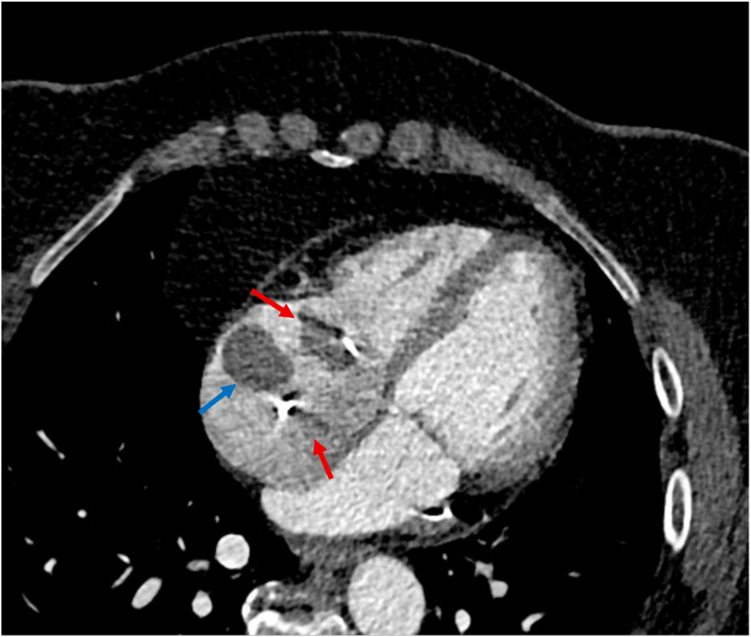
**Contrast-enhanced computed tomography scan of the aorta showing 3 hypodense filling defects (arrows) within a dilated right atrium, the largest (blue arrow) appears adherent to right atrial free wall and maximum diameter measured up to 2.9 cm.**

Eighteen months later, a CT performed for aortopathy surveillance revealed 3 new incidental filling defects within a dilated right atrium (Figure 2). Transesophageal echocardiography (TOE) subsequently confirmed large mobile echo densities (mass 1: 22 × 14 mm; mass 2: 19 × 18 mm; and mass 3: 26 × 8 mm) attached to all device leads in the right atrium (Supplemental Video 1).

There were no clinical signs or symptoms of infection or deep venous thrombosis. A CT scan of the thorax, abdomen, and pelvis revealed no evidence of malignancy. Inflammatory markers were normal, and serial peripheral blood cultures were sterile. Device function was unaffected.

**Figure 3:**
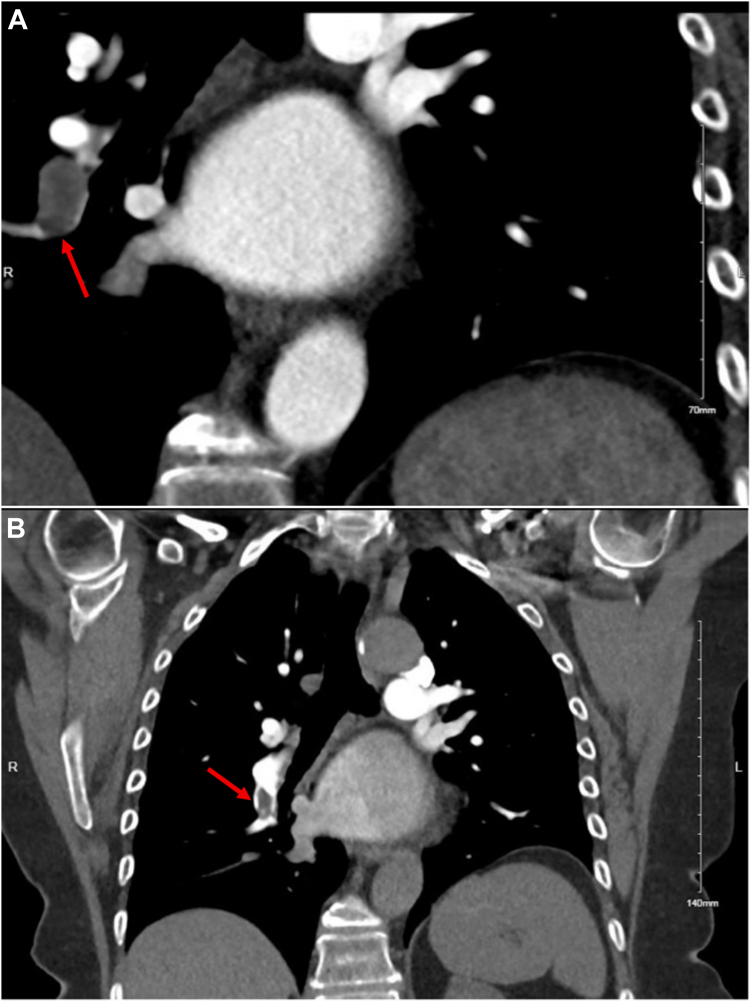
**New large filling defect in right lower lobe pulmonary artery (A).** Persistent filling defect on subsequent imaging after 4 months of antithrombotic therapy (B).

After multidisciplinary discussion, the material was felt to most likely be thrombus, and consensus was to restart anticoagulation to see if regression could be achieved medically. The right atrial masses remained unchanged in size on serial TOE after sequential 3-month trials of direct oral anticoagulation and warfarin therapy and a 2-week course of therapeutic-dose subcutaneous enoxaparin. During this time, the patient developed progressive New York Heart Association class III dyspnea. Subsequent CT imaging for aortic surveillance revealed a new large filling defect in the right lower lobe pulmonary artery and occlusion of multiple segmental vessels (Figure 3A). There was incomplete resolution after 4 months of anticoagulation (Figure 3B), raising clinical concerns regarding the risk of developing chronic thromboembolic pulmonary hypertension.

Her case was discussed again at our Heart Team meeting, and the consensus was that extraction was indicated both to confirm the diagnosis and prevent further embolization. Surgical extraction was deemed high-risk due to comorbidities, and the patient had a strong preference to avoid cardiac surgery.

**Figure 4:**
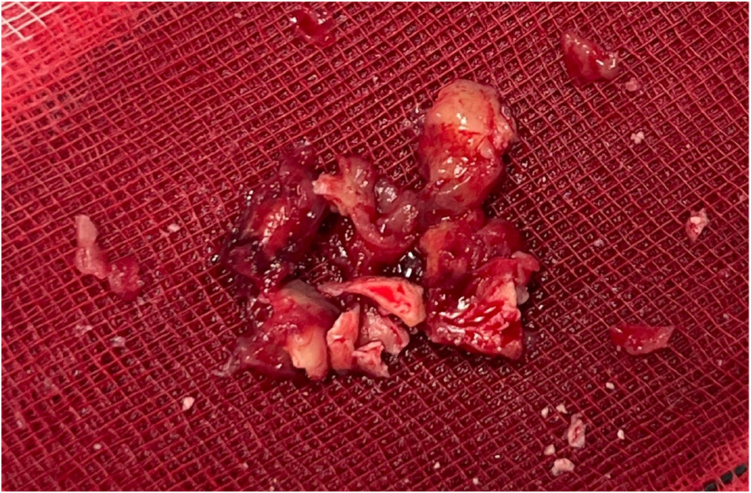
**Clinical image of aspirated material revealing multiple pieces of cream-tan fibrinous tissue measuring 35 × 26 × 5mm in aggregate.**

A percutaneous attempt was undertaken using a device with recent CE mark approval in the EU. Under general anesthesia, to allow TOE guidance, an 8F venous sheath was inserted into the right femoral vein under ultrasound guidance. A 7-cm Amplatz SuperStiff wire (Boston Scientific) was advanced to the inferior vena cava, allowing delivery of a 24F Gore DrySeal Flex (Cook Medical) introducer sheath. An AlphaVac F18^85^ aspiration thrombectomy system (AngioDynamics, Inc) was then introduced into the right atrium (Supplemental Video 2). A total of 15 aspirations were performed, guided by TOE imaging (Supplemental Video 3), with all material successfully extracted (Supplemental Video 4, Figure 4); 450 mL of extraneous blood loss was sustained. The patient remained hemodynamically stable throughout the procedure, with no immediate perioperative complications. A 0.9-unit reduction in hemoglobin (to a hemoglobin level of 11.8 g/dL) was observed on day 1 after the procedure. Transthoracic echocardiography (TTE) demonstrated no evidence of residual masses, and the tricuspid valve remained structurally and functionally unchanged from baseline (Supplemental Video 5).

**Figure 5:**
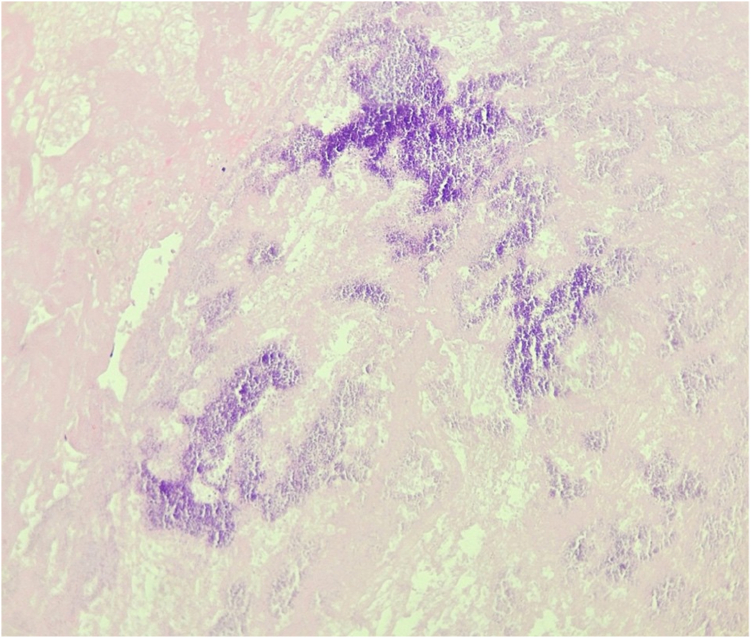
**Hematoxylin and eosin stain of aspirated material (200× magnification) revealed an organized fibrin thrombus with calcification and no evidence of external capsulation.**

The patient was discharged 2 days later on long-term warfarin, and there was no evidence of recurrent intracardiac masses on follow-up TTE at 30 days. Right ventricular function and pulmonary vascular resistance assessed by TTE were both unchanged compared to preprocedural baseline. Histopathologic analysis of the extracted material revealed a nonencapsulated organized fibrin thrombus with associated microcalcification (Figure 5). No reason was found on histopathology for the failure of 6 months of anticoagulation to resolve the material medically. Any future recurrence of thrombi may necessitate surgical device extraction and implantation of an epicardial biventricular defibrillator.

## Discussion

This case highlights a number of important learning points. First, the differential diagnosis for intracardiac masses is broad, but common causes includes infection, neoplasia, and thrombus.^1^ In this case, the presence of multiple mobile masses attached to foreign bodies in the setting of reduced ventricular function made thrombus likely, despite failure of antithrombotic therapies. The failure of response to anticoagulation was unusual and left us with a diagnostic and therapeutic conundrum, in light of her high surgical risk.

In this scenario, a definitive diagnosis can be challenging due to difficulty with tissue ascertainment, which has traditionally required surgical sampling. To the best of our knowledge, this is the first publication regarding the use of the AlphaVac F18^85^ system (AngioDynamics, Inc) for extracting right atrial masses in Europe. The device has a large funnel (33F) on a smaller shaft (18F), allowing sampling of large volumes of material without high-risk of access site problems. This case demonstrates the feasibility of a percutaneous approach using this system for extracting large right atrial masses refractory to antithrombotic therapy, which previously required the use of a perfusionist-delivered partial veno-venous extracorporeal membrane oxygenation circuit known as the AngioVac device (AngioDynamics, Inc) if a percutaneous attempt was being considered. Alternate percutaneous devices with smaller orifices may have struggled with material this large, although reports exist of their successful use.^2^^,^^3^

Finally, intracardiac thrombus can be resistant to antithrombotic therapy. A number of hypotheses may explain this phenomenon. First, organized thrombi, as in our case, are associated with platelet-mediated clot contraction and remodeling, which may create a structural barrier that reduces permeability for endogenous and exogenous fibrinolytic factors.^4^ Biochemical factors may also contribute to thrombolytic resistance. For example, inhibition of activated factor XIII, which promotes fibrin cross-linking, has been shown to amplify lysis in animal models.^5^ In our case, we expected to find a fibrotic encapsulation surrounding the masses in histology, which was not observed on microscopic analysis. However, neutrophil extracellular traps are a non-fibrin scaffold that may have contributed and are only visible with electron microscopy. These extracellular DNA fibers reduce susceptibility of thrombi to plasmin-mediated lysis, with neutrophil extracellular trap degradation found to reduce venous occlusion in preclinical studies.^6^

In summary, our case highlights the challenges for both the diagnosis and management of intracardiac thrombus, which can be resistant to anticoagulant treatment plus endogenous lysis. We demonstrate the feasibility of modern percutaneous techniques to overcome such challenges in selected cases.

## Declaration of competing interest

Andrew Sharp is a consultant to Boston Scientific, AngioDynamics, Philips, Recor Medical, and Penumbra, and holds equity in Althea Medical. The other authors reported no financial interests.

## Footnotes

To access the supplementary material accompanying this article, visit the online version of the *Journal of the Society for Cardiovascular Angiography & Interventions* at 10.1016/j.jscai.2026.104398.

## References

[1] Tyebally S., Chen D., Bhattacharyya S. (2020). Cardiac tumors: *JACC CardioOncology* state-of-the-art review. JACC CardioOncol.

[2] Moriarty J.M., Rueda V., Liao M. (2021). Endovascular removal of thrombus and right heart masses using the AngioVac system: results of 234 patients from the prospective, multicenter Registry of AngioVac Procedures in Detail (RAPID). J Vasc Interv Radiol.

[3] Clark J., Zaidi A., O’Callaghan P., von Oppell U., Sharp A.S.P. (2024). X marks the spot: catheter aspiration using the Inari FlowTriever device to debulk defibrillator lead vegetations prior to transvenous lead extraction—a case report. Eur Heart J Case Rep.

[4] Cines D.B., Lebedeva T., Nagaswami C. (2014). Clot contraction: compression of erythrocytes into tightly packed polyhedra and redistribution of platelets and fibrin. Blood.

[5] Reed G.L., Houng A.K. (1999). The contribution of activated factor XIII to fibrinolytic resistance in experimental pulmonary embolism. Circulation.

[6] Fuchs T.A., Brill A., Duerschmied D. (2010). Extracellular DNA traps promote thrombosis. Proc Natl Acad Sci U S A.

